# Comparison Between Efficacy and Safety of Remote Magnetic Navigation and Manual Catheter Navigation for Atrial Fibrillation Ablation: An Updated Meta-analysis and Systematic Review

**DOI:** 10.19102/icrm.2025.16065

**Published:** 2025-06-15

**Authors:** Rana Ijaz, Ajeet Singh, Maida Qazi, Meet Kachhadia, Laiba Qayoom, Sumaira Riaz, Hamza Nasir Chatha, Manahil Nazir, Zulekha Faisal, Muhammad Saqib, Iqra Yaseen Khan, Rimsha Bint-e-Hina, Arham Iqbal, Alina Sami Khan, Satesh Kumar, Mahima Khatri

**Affiliations:** 1 Department of Medicine, Services Institute of Medical Sciences, Lahore, Pakistan; 2 Department of Medicine, Dow University of Health Sciences, Karachi, Pakistan; 3 Dow University of Health Sciences, Karachi, Pakistan; 4 PDU Medical College, Rajkot, India; 5 Liaquat National Hospital and Medical College, Karachi, Pakistan; 6 PMAS-Arid Agriculture University, Rawalpindi, Pakistan; 7 Shifa College of Medicine, Islamabad, Pakistan; 8 Rawalpindi Medical University, Rawalpindi, Pakistan; 9 Shaheed Zulfikar Bhutto Institute of Science and Technology University, Karachi, Pakistan; 10 Chandka Medical College, SMBBMU, Larkana, Pakistan; 11 King Edward Medical University, Lahore, Pakistan; 12 Dow International Medical College, Dow University of Health Sciences, Karachi, Pakistan; 13 Department of Medicine, Shaheed Mohtarma Benazir Bhutto Medical College, Karachi, Pakistan; 14 Department of Cardiology, Dow University of Health Sciences, Karachi, Pakistan

**Keywords:** AF ablation, atrial fibrillation, fluoroscopy, manual catheter navigation, remote magnetic navigation

## Abstract

Atrial fibrillation (AF) ablation is a common treatment for symptomatic AF. Remote magnetic navigation (RMN) and manual catheter navigation (MCN) are two predominant techniques employed in this procedure, each with advantages and limitations. This meta-analysis compares the efficacy, safety, and procedural outcomes of RMN versus MCN for AF ablation. A comprehensive search was conducted across PubMed, Google Scholar, and Embase to identify relevant studies comparing RMN and MCN for AF ablation. Statistical pooling was done using Review Manager 5.4.1 (Cochrane Collaboration, London, UK). The Newcastle–Ottawa scale was used for the evaluation of bias in observational studies. We evaluated the robustness of the evidence following the guidelines outlined by the Grades of Recommendation, Assessment, Development, and Evaluation (GRADE) working group. The primary outcomes of the study included freedom from AF, procedure time, fluoroscopy time, and total complication rate in patients undergoing AF ablation either using the RMN or MCN technique. A total of 22 studies involving 5361 patients were included in the meta-analysis. The pooled analysis demonstrated comparable freedom from AF between RMN and MCN (relative risk [RR], 0.94; 95% confidence interval [CI], 0.84–1.04; *P* = .23). However, RMN was associated with a significantly prolonged procedure duration (mean difference [MD], 48.58; 95% CI, 31.49–65.66; *P* < .00001) and reduced fluoroscopy time (MD, −12.52; 95% CI, −17.84 to −7.20; *P* < .00001) compared to MCN. Additionally, RMN showed a trend toward lower total complication rates (RR, 0.63; 95% CI, 0.45–0.88; *P* = .007). In AF ablation, RMN and MCN exhibit comparable efficacy in achieving freedom from AF. However, RMN is associated with a prolonged procedure duration compared to MCN. Nonetheless, RMN offers advantages in terms of reduced fluoroscopy times and lower total complication rates, highlighting its potential for improving procedural safety. The choice between RMN and MCN should be made considering individual patient factors and procedural objectives.

## Introduction

Radiofrequency catheter ablation (RFCA) has emerged as the mainstay of therapy for patients with drug-refractory atrial fibrillation (AF) and has demonstrated more efficacy as compared to an anti-arrhythmic drug in preserving sinus rhythm throughout the medium to extended term.^1,2^ In a study of 1240 patients using an electrocardiogram (ECG) event recording system, catheter ablation was 48% more effective than drug therapy in delaying the recurrence of post-blanking AF according to an intention-to-treat analysis with death as a competing risk.^2^ Initially, 57% of participants had persistent or long-standing AF; this dropped to 26% in the drug therapy group and 16% in the catheter ablation group by the trial’s end.^2^ RFCA is indicated primarily based on the intensity of the patient’s symptoms and is applicative in patients with paroxysmal, chronic, or long-persistent AF.^1,3^ However, ablation of AF via manual catheter navigation (MCN) remains a difficult process, particularly in complex physiological or clinical scenarios. The conventional approach entails more radiation exposure and also necessitates more trained electrophysiologists.^4^ Using a magnetic field, remote magnetic navigation (RMN) regulates the mapping and ablation of the catheter inside the heart.^5^ Numerous benefits of this approach include improved stability, precision, and reduced exposure to fluoroscopy for both medical professionals and patients.^6^

RMN has been employed in the treatment of arrhythmias since 2004, exhibiting efficiency and versatility in multiple ablation techniques, including AF, ventricular tachycardia, supraventricular tachycardia, and epicardial ablation, highlighting its value in diverse clinical contexts.^4,7–11^ One of the distinctive features of the RMN technology is its ability to magnetically steer the tip of a floppy catheter, enabling the system to execute maneuvers that certain operators find challenging while using manual navigation catheters. According to the research published by Arya et al., RMN has been proven to have an exceptional safety record.^11^ In this study, the results of AF ablation employing RMN in a larger group of patients were compared with those of a similar sample of the population having manual ablation procedures.^11^ The authors reported comparable rates of success with considerably shorter fluoroscopy periods in magnetic navigation, whereas significantly longer ablation and procedure times were noted in manual navigation. Also, complications appeared to be fewer in the magnetic navigation group. According to Arya et al., among other benefits of RMN, a considerable portion of time could have also been used for different tasks while performing magnetic navigation.^11^ Although the magnetic system has substantially reduced the maximal contact forces, it also has an upgraded overall contact. As steam pops, perforations, and potentially other issues are linked to high contact forces, the safety benefit might be anticipated.^12^ Certainly, magnetic navigation has been proven to be a significant advancement in the field of technology. It is particularly effective in managing ventricular arrhythmias^9^ and can also accommodate navigation in this condition, which dominates manual navigation.

This meta-analysis endeavors to assess and compare the outcomes of RMN ablation versus MCN ablation in the treatment of AF, specifically focusing on AF recurrence. Through a systematic review approach, this study aims to identify and characterize all relevant studies that have compared RMN with MCN ablation, with a primary emphasis on evaluating the efficacy and safety of each procedure. Additionally, procedural complications and patient-reported outcomes will also be assessed to provide a comprehensive understanding of the relative merits of RMN versus MCN ablation. Ultimately, the findings of this study may inform clinical decision-making and guide future research efforts aimed at improving outcomes in patients with AF undergoing catheter ablation procedures.

## Methodology

The Preferred Reporting Items for Systematic Review and Meta-analysis guidelines were followed for this systematic review and meta-analysis.^13^

### Databases and search strategy

A systematic literature search was conducted without restriction of publication period and without using any other filters in PubMed, Google Scholar, and Embase. The literature search was performed from the above databases until May 15, 2024. To collect pertinent literature, straightforward combinations of keywords and Medical Subject Headings terms were employed, including “magnetics,” “magnets,” “magnetic phenomena,” “robotics,” “manuals,” “atrial fibrillation,” “catheters,” “catheter ablation,” “fluoroscopy,” and “pulmonary veins.” **Supplementary Table S1** presents a detailed description of the search technique employed. Additionally, the bibliography of potentially eligible articles was examined for relevant studies.

**Supplementary Table S1:** Detailed Search Strategy

Database	Search Strategy	Results
PubMed	(((((((((((((((((((((((Remote magnetic navigation) OR (RMN)) OR (Remote magnetic catheter navigation)) OR (RCN)) OR (Remote-controlled magnetic navigation)) OR (RMI)) OR (Remote magnetic system))(Magnetic navigation system)) OR (MN system)) OR (MNS)) OR (Remote magnetic navigation system)) OR (RMN system)) OR (Magnetic navigation)) OR (Robotic navigation)) OR (Remote magnetic irrigated tip ablation)) OR (Magnetic navigation ablation)) OR (Remote magnetic)) OR (Remote magnetic catheter)) OR (Remote magnetic navigation ablation)) OR (Remote ablation)) OR (Remote magnetic navigation assisted CPVI)) OR (rmtCPVI)) AND ((((((((((((((((((((((((((((((((Manual catheter navigation) OR (MCN)) OR (Manual navigation)) OR (MAN)) OR (Conventional manual-irrigated catheter catheter)) OR (CIR)) OR (Conventional manual ablation)) OR (Manual approach)) OR (Conventional approach)) OR (CON approach)) OR (Manual catheters)) OR (Conventional method)) OR (Manual steerable sheath-guided ablation)) OR (Contact force guided ablation)) OR (Contact force-sensing catheters)) OR (CF sensing catheters)) OR (CF-guided catheters)) OR (Manual catheter irrigated tip catheter ablation)) OR (Manual conventional ablation)) OR (Manual ablation)) OR (Conventional manual ablation)) OR (MAN ablation)) OR (MAN catheter ablation)) OR (CF-guided ablation)) OR (Manual navigation approach)) OR (MAN approach)) OR (Manual catheter ablation)) OR (Contact force-sensing manual catheter)) OR (Contact force-sensing ablation)) OR (MCN-CF ablation)) OR (Conventional manual pulmonary vein isolation)) OR (mCPVI))) AND (((((Atrial fibrillation) OR (AF)) OR (Paroxysmal atrial fibrillation)) OR (Paroxysmal AF)) OR (PAF)) AND ((((((((((((((((((((((Ablation) OR (Catheter ablation)) OR (Atrial fibrillation catheter ablation)) OR (AF catheter ablation)) OR (AF CA)) OR (Radiofrequency ablation)) OR (RFA)) OR (RF ablation)) OR (Radiofrequency therapy)) OR (Radio frequency ablation)) OR (Radio-frequency ablation)) OR (Radiofrequency atrial fibrillation ablation)) OR (Atrial fibrillation ablation)) OR (Percutaneous catheter ablation)) OR (Transvenous catheter ablation)) OR (Radiofrequency catheter ablation)) OR (Transvenous Electric Ablation)) OR (Electrical Catheter Ablation)) OR (Electric Catheter Ablation)) OR (Transvenous Electrical Ablation)) OR (Circumferential pulmonary vein ablation)) OR (CPVA))	158
Google Scholar	(((((((Remote magnetic navigation) OR (Remote magnetic catheter navigation)) AND (Manual catheter navigation)) OR (Manual Navigation)) AND (Atrial fibrillation)) AND (Ablation)) OR (Catheter ablation)) OR (Radiofrequency ablation)	3840
Embase	(((((((Remote magnetic navigation) OR (Remote magnetic catheter navigation)) AND (Manual catheter navigation)) OR (Manual Navigation)) AND (Atrial fibrillation)) AND (Ablation)) OR (Catheter ablation)) OR (Radiofrequency ablation)	87

*Abbreviations:* CTI, cavotricuspid isthmus; ICE, intracardiac echocardiography; PVI, pulmonary vein isolation; RMT, remote magnetic technology; WACA, wide-area circumferential ablation.

### Eligibility criteria

#### Inclusion criteria

The following criteria were used for inclusion:

1. Studies enrolling AF patients >18 years of age
2. Studies comparing the RMN group with the MCN group
3. Studies providing at least one of the primary outcomes, including freedom from AF, procedural time, fluoroscopy time, and total complication rate
4. Studies where the primary intervention was radiofrequency ablation (RFA)
5. Randomized controlled trials or non-randomized controlled studies

#### Exclusion criteria

The criteria for exclusion were as follows:

1. Studies not including AF patients >18 years of age
2. Studies not comparing the RMN group with the MCN group
3. Studies where the primary intervention was not RFA (eg, microwave ablation, cryoablation)
4. Studies not reporting at least one of the primary outcomes, including freedom from AF, procedural time, fluoroscopy time, and total complication rate
5. Case reports, case series, editorials, literature reviews, and conference abstracts

### Study selection

Titles and abstracts of all articles were screened independently by two authors (R.I. and A.I.) according to the prespecified inclusion criteria. When abstracts were not available or eligibility was unclear based on the abstract, the full papers were obtained and assessed. Some studies were excluded based on title or abstract; for all other articles, full papers were obtained and reviewed by the same two authors, with each assessor blinded to the decision of the other. Discrepancies in assessing studies as eligible were resolved by mutual agreement or by a third-party arbitrator (S.K.). We identified duplicate publications by reviewing the study name, author, study population, and study dates. The articles meeting the inclusion criteria and passing both stages of screening (title/abstract and full text) were selected for the meta-analysis.

### Data extraction

Published data were extracted using Google Sheets for each study independently by two authors (S.R. and H.N.C.) and cross-matched by two other authors. Disagreements were resolved by consensus, by a third-party arbitrator, or by attempting to contact the authors of the study. The data sheet included: (1) the name of the first author, along with the year of publication; (2) the demographic characteristics of the included participants, if available, for both the RMN and MCN groups, including the number of participants, age, sex distribution, and body mass index (BMI) values; (3) types of AF (paroxysmal and persistent or permanent); (4) incidence rates of diabetes mellitus (DM), hypertension (HTN), cerebrovascular accidents (CVAs), and coronary artery disease (CAD); (5) left atrial size or diameter; (6) prior ablation; (7) follow-up time (months); (8) primary outcomes of the studies, including freedom from AF, total procedure time, fluoroscopy time, and total complication rate; and (9) secondary outcomes if available, including acute success rate, pericardial effusion with or without tamponade, vascular access complications, AF recurrence rate, ablation time, and radiofrequency (RF) application duration. For continuous outcome data, we extracted the mean (standard deviation) values. Standardized statistical conversions were made if the data were reported as median (interquartile range). Dichotomous data were extracted in events/total format. Graphical data were extracted using the online application PlotDigitizer (Plot Digitizer, Phoenix, AZ, USA). The extracted data were verified by a third reviewer (R.I.).

### Quality assessment and Grades of Recommendation, Assessment, Development, and Evaluation strength of evidence

The risk of bias in each included study was assessed independently by two reviewers (R.I. and M.N.) using the Newcastle–Ottawa scale (NOS) for quality assessment, which assesses the risk of bias in observational studies.^14^ The studies are judged by the NOS on three broad perspectives using a 9-point scale, as follows: (1) the selection of study groups (0–4 points), (2) comparability (0–2 points), and (3) exposure or outcome (0–3 points). Disagreements in scoring were resolved through discussion. The methodological quality of the studies was classified as low risk of bias (0–3 points), moderate risk of bias (4–6 points), or high risk of bias (7–9 points).

For each statistically pooled outcome, we assessed the overall strength of the evidence using the guidelines created by the Grades of Recommendation, Assessment, Development, and Evaluation (GRADE) working group.^15^ Following this, the evidence strength was classified as high quality, moderate quality, low quality, or very low quality.

### Statistical analysis

The statistical analysis for this meta-analysis was carried out using Review Manager version 5.4.1 (Cochrane Collaboration, London, UK). The results are shown using forest plots, which depict the pooled effect of relative risks (RRs) for dichotomous outcomes and weighted mean differences (MDs) for continuous outcomes. To ensure the precision of the findings, a random-effects model using the generic inverse variance approach was applied. *P* < .05 was defined as the threshold for statistical significance.

The results of pooled studies are demonstrated in the forest plots, and funnel plots were created to evaluate publication bias. To confirm our findings, Egger’s regression test was applied. Higgin’s *I*^2^ test was used to assess the heterogeneity levels. The degree of heterogeneity was defined as low (*I*^2^ < 25%), moderate (*I*^2^ = 25%–75%), or high (*I*^2^ > 75%). Moderate and high heterogeneity necessitated the exploration of the causes of heterogeneity.^16^

### Methods for exploring the sources of heterogeneity

Sensitivity analysis, subgroup analysis, and meta-regression were conducted to investigate potential explanations for significant heterogeneity. A sensitivity analysis was performed to assess the contribution of each study to the pooled estimate by excluding individual trials one at a time and recalculating the pooled estimate for the remaining studies (leave-one-out meta-analysis).^16^ To perform subgroup analysis, the studies were divided into two subgroups according to the following conditions: case–control and cohort, age <60 years and >60 years, presence and absence of DM, presence and absence of HTN, presence and absence of CAD, and prior ablation and prior ablation not done, respectively. A univariate meta-regression was performed using the Comprehensive Meta-Analysis software (Biostat, Inc., Englewood, NJ, USA) to identify variables that might have been a possible source of substantial heterogeneity across the results of four primary outcomes, including freedom from AF, procedure time, fluoroscopy time, and total complication rate.

## Results

### Eligible studies

**Figure 1:**
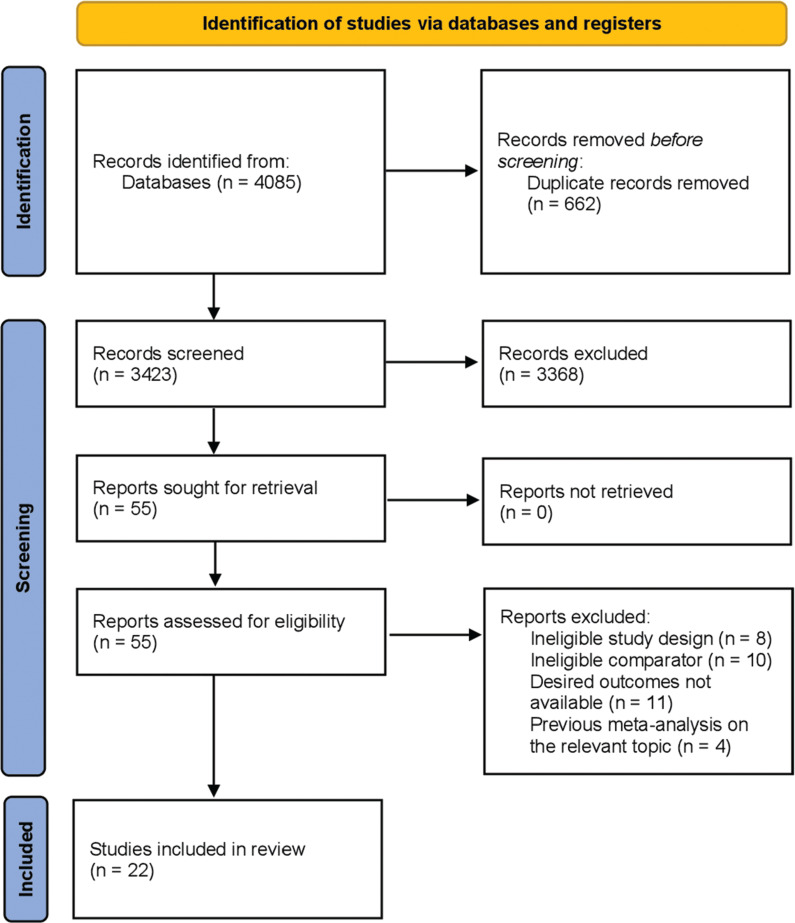
Preferred Reporting Items for Systematic Review and Meta-analysis flow diagram demonstrating the search strategy and study selection process for the meta-analysis. Initially, 4085 articles were retrieved, followed by a review to identify duplicates and assess them based on their titles and abstracts. Ultimately, 22 observational studies fulfilled the inclusion criteria for the analysis.

Out of the 4085 studies initially identified through our search, we retained 3423 unique papers after removing duplicates. Subsequently, upon reviewing the titles and abstracts, 3368 articles were excluded as they were deemed irrelevant or did not meet the eligibility criteria. The full texts of the remaining 55 citations were meticulously examined, leading to the further exclusion of 33 studies that did not meet the inclusion criteria. Finally, our analysis included 22 observational studies^4,6,11,17–35^ that met the criteria. **Figure 1** illustrates the detailed process of study selection.

### Baseline characteristics of included patients

We incorporated 22 non-randomized controlled studies (17 cohort observational and 5 case–control observational studies). **Table 1** displays the baseline characteristics observed in the included studies. In total, our review included 5361 patients (2285 patients in the RMN group and 3076 patients in the MCN group), with study sample sizes ranging from 21 to 667 individuals. Both groups showed comparability regarding mean age, mean BMI, type of AF, and mean left atrial size or volume across the included studies. We additionally compiled the follow-up data from the studies included in our analysis. Among the 22 included studies, 14 studies reported on patients with HTN, 11 studies included those with a history of DM, 9 studies involved patients with a history of CAD, 6 studies included individuals with prior ablation, and 4 studies reported on patients with a history of CVA. The ablation characteristics, ablation lesion design, and ablation settings of the included studies are summarized in **Supplementary Table S2**.

**Table 1:** Baseline Characteristics of Included Studies

Study/Year	Sample Size (n)	Mean Age (Years)		Male Sex (%)		Mean BMI (kg/m^2^)		AF Type (n)				DM (%)		HTN (%)		CVA (%)		CAD (%)		Mean Left Atrial Diameter (mm) or Size (mL)		Prior Ablation (n)		Mean Follow-up Time (Months)	
								Paroxysmal AF		Persistent or Permanent AF															
		RMN	MCN	RMN	MCN	RMN	MCN	RMN	MCN	RMN	MCN	RMN	MCN	RMN	MCN	RMN	MCN	RMN	MCN	RMN	MCN	RMN	MCN	RMN	MCN
Katsiyiannis et al. (2008)^4^	20/20	—	—	—	—	—	—	13	14	7	6	—	—	—	—	—	—	—	—	46.1	45.3	—	—	12.0	12.0
Lim et al. (2017)^6^	214/229	54.9	54.0	74.6	77.1	—	—	161	154	53	75	—	—	—	—	—	—	—	—	—	—	42	37	—	—
Arya et al. (2011)^11^	70/286	57.9	58.0	65.7	66.1	—	—	35	216	35	70	18.6	12.2	77.1	53.8	—	—	—	—	40.5	42.8	21	84	6.0	6.0
Pappone et al. (2006)^17^	40/28	55.9	56.0	60	57.1	—	—	25	16	15	12	7.5	10.7	30	28.6	—	—	—	—	42.2	41.4	9	6	—	—
Kim et al. (2008)^18^	75/91	—	—	—	—	—	—	—	—	—	—	—	—	—	—	—	—	—	—	—	—	—	—	—	—
Miyazaki et al. (2010)^19^	30/44	60.2	57.6	76.6	84.1	—	—	30	44	—	—	—	—	—	—	—	—	—	—	42.0	40.7	—	—	14.0	15.0
Sorgente et al. (2010)^20^	35/29	55.7	56.1	83.3	89.7	26.37	26.48	24	20	6	9	0	3.4	40	58.6	0	3.4	16.7	6.9	41.1	42.4	—	—	11.8	10.9
Lüthje et al. (2011)^21^	107/54	62.0	61.0	61.7	63	—	—	34	18	73	36	—	—	70	74	—	—	22	13	47.0	45.0	23	12	12.0	12.0
Solheim et al. (2011)^22^	23/65	59.0	57.0	96	79	—	—	15 irrigated catheter; 14 non-irrigated catheter	40	8 irrigated catheter; 12 non-irrigated catheter	25	—	—	—	—	—	—	—	—	—	—	7	18	12.2	12.2
Bauernfeind et al. (2011)^23^	56/76	48.0	52.0	—	—	—	—	0	60	56	16	—	—	—	—	—	—	—	—	—	—	—	—	15.0	14.0
Choi et al. (2011)^24^	41/70	57.0	55.0	75	77	25.0	25.0	24	43	17	27	14	13	36	42	—	—	9	10	43.7	41.2	—	—	3.0	3.0
Akca et al. (2013)^25^	33/34	49.6	52.8	—	—	—	—	25	23	8	11	—	—	—	—	—	—	—	—	43.1	44.9	—	—	19.0	19.0
Koutalas et al. (2015)^26^	70/70	59.5	56.89	65.7	71.4	—	—	35	35	35	35	18.6	18.6	77	42.8	—	—	7.1	11.4	41.3	45.6	21	32	30.34	27.32
Akca et al. (2015)^27^	108/306	48.4	51.7	—	—	—	—	—	—	—	—	—	—	—	—	—	—	—	—	—	—	—	—	19.0	19.0
Weiss et al. (2016)^28^	315/312	66.1	64.1	64.4	63.8	30.8	31.3	182	167	133	145	22.2	28.8	62.5	52.9	2.5	2.9	35.2	38.5	—	—	—	—	36.0	36.0
Adragão et al. (2016)^29^	287/287	58.3	57.9	70	67.6	27.7	27.6	213	207	74	80	—	—	38	39	—	—	—	—	—	—	—	—	31.2	31.2
Kataria et al. (2017)^30^	114/222	61.3	59.8	65.7	61.2	—	—	114	222	—	—	9.6	7.6	31	27	—	—	—	—	40.7	39.6	—	—	27.2	27.2
Bun et al. (2017)^31^	11/10	60.5	60.4	72	80	—	—	11	10	—	—	0	0	36	20	9	0	36	0	116.0 mL	109.0 mL	—	—	21.0	21.0
Yuan et al. (2017)^32^	112/102	60.7	60.1	67.9	70.6	—	—	78	72	34	30	4.5	6.9	37.5	38.2	3.6	4.9	7.1	8.8	76.4 mL	76.9 mL	—	—	39.0	44.0
Jez et al. (2020)^33^	57/89	63.6	61.7	71.9	76.4	29.08	29.60	30	43	27	46	26.31	20.22	56.14	50.56	—	—	—	—	47.58	47.36	—	—	6.0	6.0
Schlögl et al. (2022)^34^	287/380	62.7	62.1	60	65	28.5	28.9	105	160	181	221	10	12	74	78	—	—	18	21	46.6	45.8	—	—	26.3	28.6
Schlögl et al. (2024)^35^	180/272	63.4	64.0	61	61	27.8	29.3	66	86	114	186	15	14	78	82	—	—	25	22	46.8	45.6	—	—	18.8	18.7

*Abbreviations:* AF, atrial fibrillation; BMI, body mass index; CAD, coronary artery disease; CVA, cerebrovascular accident; DM, diabetes mellitus; HTN, hypertension; MCN, manual catheter navigation; RMN, remote magnetic navigation.

**Supplementary Table S2:** Overview of Ablation Features

Studies	Year	System	Ablation Catheter				Circular Mapping Catheter	ICE Usage
			RMN		MCN			
Katsiyiannis et al.^4^	2008	Niobe II	4-mm tip magnetic Celsius		8-mm tip Blazer		Yes (Lasso)	Not mentioned
Lim et al.^6^	2017	Niobe II	3.5-mm irrigated Navistar RMT ThermoCool		3.5-mm Navistar ThermoCool or Flexibility or Tacticath		Yes (Lasso)	Yes
Arya et al.^11^	2011	The Niobe Stereotaxis MNS	8-mm Navistar RMT or 3.5-mm irrigated tip Navistar RMT ThermoCool		Irrigated tip CoolPath		Yes (Inquiry Optima)	Not mentioned
Pappone et al.^17^	2006	Niobe II	4-mm tip Navistar RMT		Not mentioned		Not mentioned	Not mentioned
Kim et al.^18^	2008	Niobe II	CARTO: 4-mm or 8-mm tip Navistar RMT; ESI/NavX or no mapping: 4-mm tip Celsius		4-mm or 8-mm tip EPT		Not mentioned	Not mentioned
Miyazaki et al.^19^	2010	The Niobe Stereotaxis MNS	3.5-mm externally irrigated magnetic tip ThermoCool RMT		3.5-mm externally irrigated tip		Yes (Lasso)	Not mentioned
Sorgente et al.^20^	2010	Niobe II	3.5-mm irrigated ThermoCool RMT Navistar		3.5-mm irrigated tip Navistar ThermoCool		Yes (Lasso)	Not mentioned
Lüthje et al.^21^	2011	Niobe II	3.5-mm open-irrigated Navistar ThermoCool RMT		3.5-mm open-irrigated Navistar ThermoCool		Not mentioned	Not mentioned
Solheim et al.^22^	2011	Niobe II	Navistar RMT ThermoCool or Navistar Celsius RMT		Irrigated RFA ThermoCool or Navistar ThermoCool		Yes (Lasso or Optima)	Not mentioned
Bauernfeind et al.^23^	2011	Niobe II	Navistar RMT ThermoCool		Navistar ThermoCool		Yes (Lasso)	Yes
Choi et al.^24^	2011	The Niobe Stereotaxis MNS	3.5-mm irrigated Navistar ThermoCool RMT		3.5-mm Navistar ThermoCool		Yes (Lasso)	Not mentioned
Akca et al.^25^	2013	Niobe II	Navistar RMT ThermoCool		Navistar ThermoCool		Not mentioned	Yes
Koutalas et al.^26^	2015	The Niobe Stereotaxis MNS	3.5-mm irrigated tip Navistar RMT ThermoCool		3.5-mm irrigated tip CoolPath or Navistar ThermoCool		Yes (Inquiry Optima)	Not mentioned
Akca et al.^27^	2015	Niobe II	Not mentioned				Not mentioned	Yes
Weiss et al.^28^	2016	The Niobe II and Epoch	3.5-mm open-irrigated Navistar ThermoCool RMT		3.5-mm open-irrigated Navistar ThermoCool or 3.5-mm open–force-sensing irrigated ThermoCool SmartTouch		Yes (Lasso)	Yes
Adragão et al.^29^	2016	Niobe II	Irrigated catheter (without details)				Yes (without details)	Not mentioned
Kataria et al.^30^	2017	The Niobe/Epoch	3.5-mm irrigated tip Navistar RMT ThermoCool		3.5-mm open-irrigated tip Navistar ThermoCool or Navistar ThermoCool SmartTouch		Yes (Lasso)	Not mentioned
Bun et al.^31^	2017	Odyssey Stereotaxis	3.5-mm open-irrigated, magnetic ablation catheter (Navistar ThermoCool RMT)		Irrigated CF sensing catheter (ThermoCool SmartTouch)		Yes (Lasso)	Not mentioned
Yuan et al.^32^	2017	Niobe ES/Epoch	3.5-mm irrigated tip ThermoCool/C3/RMT		3.5-mm irrigated tip Navistar ThermoCool or contact force catheter SmartTouch		Yes (Lasso)	Not mentioned
Jez et al.^33^	2020	Niobe ES/EAM	Navistar ThermoCool RMT		Ensite Velocity and TactiCath Quartz catheter with direct contact force measurement		Yes	Not mentioned
Schlögl et al.^34^	2022	Niobe II Stereotaxis	3.5-mm open-irrigated, magnetic mapping and ablation catheter (Navistar ThermoCool RMT)		Either a manually guided 3.5-mm open-irrigated tip mapping and ablation catheter (Navistar ThermoCool) or a manually guided 3.5-mm open-irrigated tip Surround Flow mapping and ablation catheter (Navistar ThermoCool SF)		Yes	Not mentioned
Schlögl et al.^35^	2024	Niobe II Stereotaxis	3.5-mm open-irrigated, magnetic mapping and ablation catheter (Navistar ThermoCool RMT)		Open-irrigated tip and contact force-sensing mapping and ablation catheter (Thermocool SmartTouch Surround Flow)		Yes	Not mentioned
**Studies**	**Year**	**Ablation Lesion Design**	**Temperature (°C)**		**Power (W)**		**Flow Rate (mL/min)**	
			**RMN**	**MCN**	**RMN**	**MCN**	**RMN**	**MCN**
Katsiyiannis et al.^4^	2008	PVI, WACA + linear lesions from the left inferior pulmonary vein to the mitral valve + across the anterior left atrial roof	50	55	30	45	—	—
Lim et al.^6^	2017	PVI + superior vena cava + CTI + CFAE	40		40		—	—
Arya et al.^11^	2011	PVI + linear ablations between the circular lesions along the roof and posterior wall of the left atrium (box lesion) + between the circular lesion and the mitral annulus	48		35		30	
Pappone et al.^17^	2006	PVI + mitral isthmus ablation line	65	—	50	—	—	—
Kim et al.^18^	2008	PVI	—	—	—	—	—	—
Miyazaki et al.^19^	2010	PVI + CTI	45		35		30	
Sorgente et al.^20^	2010	PVI	48		35	—	—	—
Lüthje et al.^21^	2011	PVI	45		40 (30 for the posterior wall)		30 (17 for the posterior wall)	
Solheim et al.^22^	2011	PVI + CTI + additional ablation between the two PVs if needed + two lines connecting the two contralateral superior and inferior veins + CAFE + combinations	55	50	40	30–35	20	15–20
Bauernfeind et al.^23^	2011	PVI + linear ablation in the left and/or right atrium	—	—	—	—	—	—
Choi et al.^24^	2011	PVI, antral ablation with carina, roof and posterior lines + anterior ablation line, connected roof line, and anterior mitral annulus through the medial side of the left atrial appendage	39		40	35	—	—
Akca et al.^25^	2013	PVI + linear ablations in the first roof line and mitral line + postero-inferior line + ablation in the LA and coronary sinus	—	—	—	—	—	—
Koutalas et al.^26^	2015	PVI + linear ablations between the left and right PVs (box lesion) at the posterior wall + between the inferior borders of the left inferior PV to the lateral mitral annulus	48		35		30	
Akca et al.^27^	2015	Not mentioned	—	—	—	—	—	—
Weiss et al.^28^	2016	PVI	—	—	40–50 (25–35 for the posterior wall)	30–40 (20–30 for the posterior wall)	—	—
Adragão et al.^29^	2016	PVI + CTI	—	—	—	—	—	—
Kataria et al.^30^	2017	PVI	48		20–35		20 mL/min	
Bun et al.^31^	2017	PVI + a 6-F steerable decapolar catheter positioned in the coronary sinus	42	42	30 and 40 at the posterior left atrial wall		17 mL/min and 30 mL/min	
Yuan et al.^32^	2017	PVI + linear lesions	48		30–35 for the posterior wall,35–40 for other areas		—	—
Jez et al.^33^	2020	PVI + catheter via the femoral vein into the coronary sinus + LA, linear lesions on the roof and mitral isthmus			Anterior wall ablation at 40 W and posterior wall ablation at 35 W	Anterior wall ablation at 30 W and posterior wall ablation at 25 W		
Schlögl et al.^34^	2022	PVI + a 6-F steerable decapolar catheter positioned in the coronary sinus via femoral veins	45	45	30–40 at the posterior LA Wall		17 mL/min or 30 mL/min	
Schlögl et al.^35^	2024	PVI + a 6-F steerable decapolar catheter positioned in the coronary sinus via femoral veins	45		30 or 40 at the posterior LA wall		17 mL/min or 30 mL/min	

*Abbreviations:* CAFE, complex atrial fractionated electrogram; CTI, cavotricuspid isthmus; ICE, intracardiac echocardiography; LA, left atrium; MCN, manual catheter navigation; PV, pulmonary vein; PVI, pulmonary vein isolation; RMT, remote magnetic technology; WACA, wide area circumferential ablation. All catheters are from Biosense Webster (Diamond Bar, CA, USA).

### Quality assessment and publication bias

**Supplementary Figure S1:**
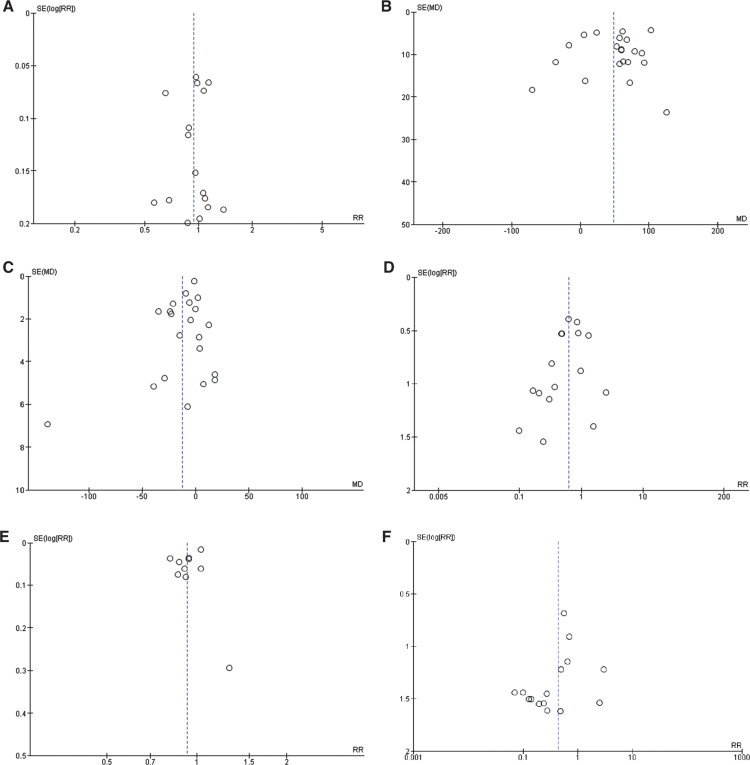

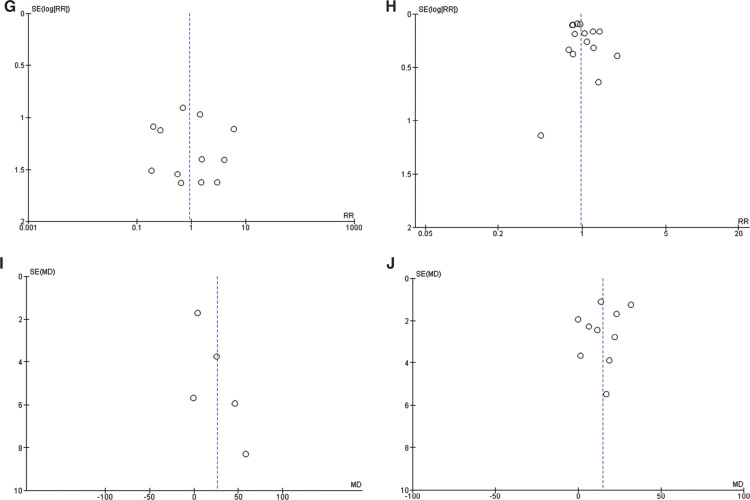
Funnel plots of primary and secondary outcomes. **A:** Freedom from atrial fibrillation. **B:** Procedure time. **C:** Fluoroscopy time. **D:** Total complication rate. **E:** Acute success rate. **F:** Pericardial effusion with or without tamponade. **G:** Vascular access complications. **H:** Atrial fibrillation recurrence rate. **I:** Ablation time. **J:** Radiofrequency application duration. *Abbreviations:* AF, atrial fibrillation; RF, radiofrequency.

We assessed the quality of our included studies using the NOS. Based on the NOS criteria, 1 observational study out of the 22 assessed was rated as moderate quality, while the rest were classified as high quality, as displayed in **Supplementary Table S3**. The funnel plots demonstrated that the results were not influenced by publication bias, as illustrated in **Supplementary Figure S1**.

**Supplementary Table S3:** Quality Assessment of Observational Studies Using the Newcastle–Ottawa Scale

Case–Control Study									

Studies	Selection				Comparability	Exposure			Total
	Is the Case Definition Adequate?	Representativeness of the Cases	Selection of Controls	Definition of Controls	Comparability of Cases and Controls on the Basis of Design or Analysis	Ascertainment of Exposure	Same Method of Ascertainment for Cases and Controls	Non-response Rate	
Lim et al. (2017)^6^	1	1	0	1	1	1	1	1	7
Arya et al. (2011)^11^	1	1	0	1	1	1	1	1	7
Pappone et al. (2006)^17^	1	1	0	1	1	1	1	1	7
Kim et al. (2008)^18^	1	1	0	1	1	1	1	1	7
Koutalas et al. (2015)^26^	1	1	0	1	2	1	1	1	8
Cohort Study									

**Studies**	**Selection**				**Comparability**	**Outcome**			**Total**
	**Representativeness of the Exposed Cohort**	**Selection of the Non-exposed Cohort**	**Ascertainment of Exposure**	**Demonstration That Outcome of Interest Was Not Present at Start of Study**	**Comparability of Cohorts on the Basis of Design or Analysis**	**Assessment of Outcome**	**Was Follow-up Long Enough for Outcomes to Occur**	**Adequacy of Follow-up of Cohorts**	
Katsiyiannis et al. (2008)^4^	1	0	1	1	0	1	1	1	6
Miyazaki et al. (2010)^19^	1	1	1	1	1	1	1	1	8
Sorgente et al. (2010)^20^	1	1	1	0	1	1	1	1	7
Lüthje et al. (2011)^21^	1	1	1	1	2	1	1	1	9
Solheim et al. (2011)^22^	1	1	1	1	1	1	1	1	8
Bauernfeind et al. (2011)^23^	1	1	1	1	1	1	1	1	8
Choi et al. (2011)^24^	1	1	1	1	1	1	0	1	7
Akca et al. (2013)^25^	1	1	1	1	2	1	1	1	9
Akca et al. (2015)^27^	1	1	1	1	1	1	1	1	8
Weiss et al. (2016)^28^	1	1	1	0	1	1	1	1	7
Adragão et al. (2016)^29^	1	1	1	1	1	1	1	0	7
Kataria et al. (2017)^30^	1	1	1	0	1	1	1	0	6
Bun et al. (2017)^31^	1	0	1	1	1	1	1	1	7
Yuan et al. (2017)^32^	1	1	1	0	1	1	1	1	7
Jez et al. (2020)^33^	1	1	1	1	2	1	1	1	9
Schlögl et al. (2022)^34^	1	1	1	1	2	1	1	1	9
Schlögl et al. (2024)^35^	1	1	1	1	2	1	1	1	9

*Abbreviation:* NOS, Newcastle–Ottawa scale.

### Outcomes

The outcomes are depicted in a tabulated manner in **Tables 2 and 3**.

**Table 2:** Summary of Quantitative Analysis and Heterogeneity Analysis in Primary Outcomes

Outcomes	No. of Studies	Quantitative Data Synthesis					Heterogeneity Analysis
		RR	MD	95% CI	*Z* Value	*P* Value	*I*^2^ (%)
Freedom from atrial fibrillation	16	0.94	—	0.84 to 1.04	1.20	.23	73
Procedure time	21	—	48.58	31.49 to 65.66	5.57	<.00001	96
Fluoroscopy time	21	—	−12.52	−17.84 to −7.20	4.61	<.00001	99
Total complication rate	16	0.63	-	0.45 to 0.88	2.71	.007	0

*Abbreviations:* CI, confidence interval; MD, mean difference; RR, relative risk.

**Table 3:** Summary of Quantitative Analysis and Heterogeneity Analysis in Secondary Outcomes

Outcomes	No. of Studies	Quantitative Data Synthesis					Heterogeneity Analysis
		RR	MD	95% CI	*Z* Value	*P* Value	*I*^2^ (%)
Acute success rate	10	0.93	—	0.86–1.01	1.83	.07	87
Pericardial effusion with or without tamponade	15	0.44	—	0.23–0.82	2.58	.010	0
Vascular access complications	12	0.93	—	0.46–1.89	0.19	.85	0
Atrial fibrillation recurrence rate	15	0.97	—	0.88–1.07	0.54	.59	17
Ablation time	5	—	25.80	7.28–44.32	2.73	.006	96
Radiofrequency application duration	10	—	14.67	7.58–21.76	4.05	<.0001	97

*Abbreviations:* CI, confidence interval; MD, mean difference; RR, relative risk.

#### Primary outcomes

The co-primary outcomes analyzed in our study were freedom from AF, procedure time, fluoroscopy time, and total complication rate.

##### Freedom from atrial fibrillation

**Figure 2:**
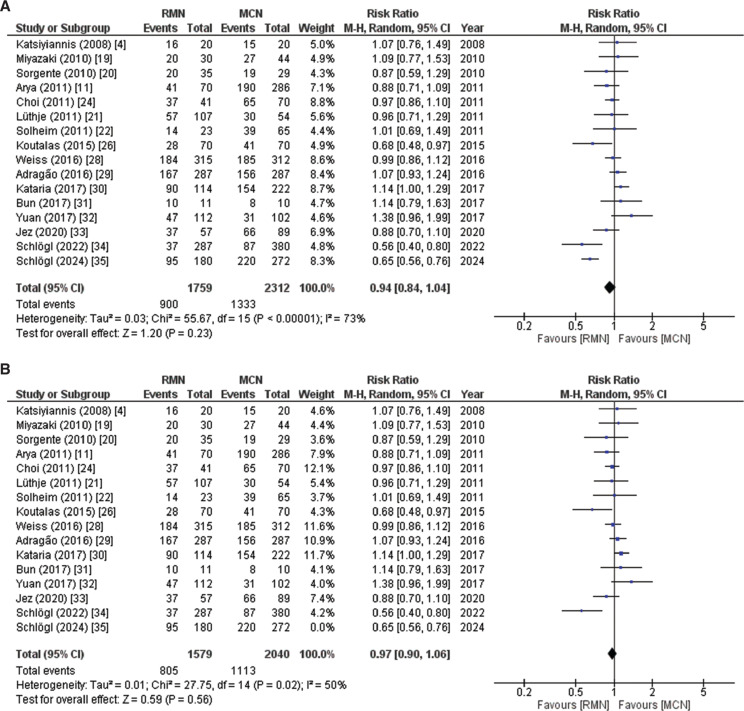
**A:** Forest plot of freedom from atrial fibrillation (AF). This figure summarizes a pooled analysis of 16 studies (4071 patients) on freedom from AF, showing no significant difference between remote magnetic navigation and manual catheter navigation procedures. **B:** Forest plot of freedom from AF with sensitivity analysis. This figure shows a sensitivity analysis of freedom from AF, with a reduction in heterogeneity from 73% to 50% achieved by excluding Schlögl et al.^35^ *Abbreviations:* AF, atrial fibrillation; CI, confidence interval; M–H, Mantel–Haenszel; MCN, manual catheter navigation; RMN, remote magnetic navigation; RR, relative risk.

Freedom from AF typically refers to the absence of AF episodes lasting longer than a defined period of time (often 30 s or 1 min) after a blanking period following the ablation procedure. Sixteen studies including 4071 patients (RMN, 1759; MCN, 2312) reported freedom from AF between 3- and 44-month follow-up. The pooled analysis indicated that this outcome was associated with a higher number of events in the MCN group compared to the RMN group, but there was no statistically significant difference in terms of freedom from AF between the patients who underwent the RMN procedure and the control group treated with the MCN method (RR, 0.94; 95% confidence interval [CI], 0.84–1.04; *P* = .23), as illustrated in **Figure 2A**. Due to high heterogeneity (*I*^2^ = 73%), a leave-one-out sensitivity analysis was conducted, which revealed that removing the study by Schlögl et al. published in 2024^35^ reduced the heterogeneity to *I*^2^ = 50%, as displayed in **Figure 2B**. The GRADE strength of the evidence was very low, as depicted in **Supplementary Table S4**. The absolute success rates ranged from 43% to 100% in the RMN group and from 34% to 100% in the MCN group, reflecting variability in ablation strategies, patient characteristics, and outcome measures across studies.

**Supplementary Table S4:** Summary of Findings

Outcomes	No. of Participants (Studies)	Certainty of the Evidence (GRADE)	Relative Effect (95% CI)	Anticipated Absolute Effects	
				Risk with MCN	Risk Difference with RMN
Freedom from atrial fibrillation	4071 (16 non-randomized studies)	⨁◯◯◯ Very low	RR 0.94^a^ (0.84–1.04)	577 per 1000	35 fewer per 1,000^a^ (92 fewer to 23 more)
Procedure time	4734 (21 non-randomized studies)	⨁◯◯◯ Very low	—	The mean total procedure time was 0 min^a^	MD 48.58 higher (31.49 higher to 65.66 higher)
Fluoroscopy time	5215 (21 non-randomized studies)	⨁◯◯◯ Very low	—	The mean fluoroscopy time was 0 min^a^	MD 12.52 lower (17.84 lower to 7.2 lower)
Total complication rate	4945 (16 non-randomized studies)	⨁⨁◯◯ Low	RR 0.63^a^ (0.45–0.88)	45 per 1000	17 fewer per 1000^a^ (25 fewer to 5 fewer)
Acute success rate	2239 (10 non-randomized studies)	⨁⨁◯◯ Low	RR 0.93^a^ (0.86–1.01)	923 per 1000	65 fewer per 1000^a^ (129 fewer to 9 more)
Pericardial effusion	4517 (15 non-randomized studies)	⨁⨁⨁⨁ High	RR 0.44^a^ (0.23–0.82)	21 per 1000	12 fewer per 1000^a^ (16 fewer to 4 fewer)
Vascular access complications	3827 (12 non-randomized studies)	⨁⨁◯◯ Low	RR 0.93^a^ (0.46–1.89)	10 per 1000	1 fewer per 1000^a^ (5 fewer to 9 more)
Atrial fibrillation recurrence rate	3996 (15 non-randomized studies)	⨁⨁◯◯ Low	RR 0.97^a^ (0.88–1.07)	339 per 1000	10 fewer per 1000^a^ (41 fewer to 24 more)
Ablation time	677 (5 non-randomized studies)	⨁◯◯◯ Very low	—	The mean ablation time was 0 min^a^	MD 25.8 higher (7.28 higher to 44.32 higher)
Radiofrequency application duration	3195 (10 non-randomized studies)	⨁◯◯◯ Very low	—	The mean radiofrequency application duration was 0 min^a^	MD 14.67 higher (7.58 higher to 21.76 higher)

*Abbreviations:* CI, confidence interval; GRADE, Grades of Recommendation, Assessment, Development, and Evaluation; MCN, manual catheter navigation; MD, mean difference; RMN, remote magnetic navigation; RR, relative risk. ^a^The risk in the intervention group (and its 95% CI) is based on the assumed risk in the comparison group and the relative effect of the intervention (and its 95% CI).

GRADE Working Group grades of evidence:
High certainty: We are very confident that the true effect lies close to that of the estimate of the effect. Moderate certainty: We are moderately confident in the effect estimate; the true effect is likely to be close to the estimate of the effect, but there is a possibility that it is substantially different. Low certainty: Our confidence in the effect estimate is limited; the true effect may be substantially different from the estimate of the effect. Very low certainty: We have very little confidence in the effect estimate; the true effect is likely to be substantially different from the estimate of effect.

**Supplementary Table S5:** Egger’s Regression Test

Primary Outcomes	Egger’s Test	
	*P* Value	*t* Value
Freedom from atrial fibrillation	.76	0.32
Procedure time	.98	0.02
Fluoroscopy time	.74	0.34
Total complication rate	.15	1.53

To account for heterogeneity, subgroup analyses were performed based on follow-up duration, monitoring methods, and patient characteristics (eg, paroxysmal versus persistent AF). Notably, a subgroup of studies with rigorous follow-up (eg, 12-month Holter monitoring) showed no difference in recurrence rates between RMN and MCN groups.^20–22,26,28,29,32^ This underscores the importance of standardized monitoring methods in future studies to ensure accurate and comparable assessment of ablation success.

##### Procedure time

**Figure 3:**
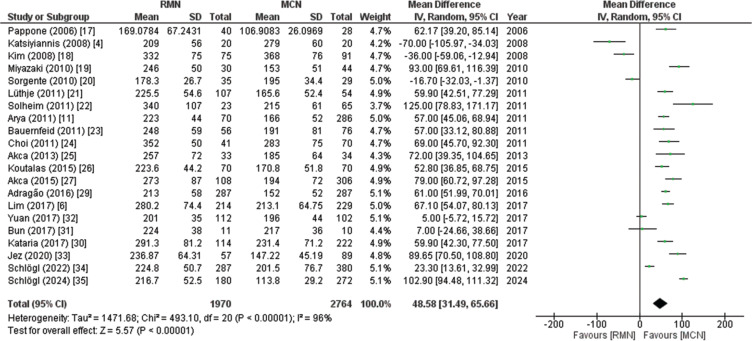
Forest plot of procedure time. This figure shows a pooled analysis of 21 studies, indicating that remote magnetic navigation prolonged procedure times by 48.58 min compared to manual catheter navigation. *Abbreviations:* CI, confidence interval; IV, inverse variance; MCN, manual catheter navigation; MD, mean difference; RMN, remote magnetic navigation; SD, standard deviation.

Twenty-one studies including 4734 patients (RMN, 1970; MCN, 2764) reported the procedure time. The pooled analysis revealed that the procedure time of patients treated with the RMN system was significantly longer than that of the control group treated with the MCN technique. The use of RMN increased the procedure time by a mean duration of 48.58 min (MD, 48.58; 95% CI, 31.49–65.66; *P* < .00001), as illustrated in **Figure 3**. Due to high heterogeneity (*I*^2^ = 96%), a leave-one-out sensitivity analysis was performed, and the result seemed to be non-significant. The subgroup analysis revealed that the procedure time was significantly prolonged (*P* = .04) in patients with prior AF ablation (MD, 70.26; 95% CI, 54.01–86.50; *P* < .00001) compared to those without prior ablation procedures (MD, 40.50; 95% CI, 17.25–63.76; *P* = .0006), demonstrating that prior AF ablation is a source of heterogeneity in the included studies. The GRADE strength of the evidence was very low, as displayed in **Supplementary Table S4**.

##### Fluoroscopy time

**Figure 4:**
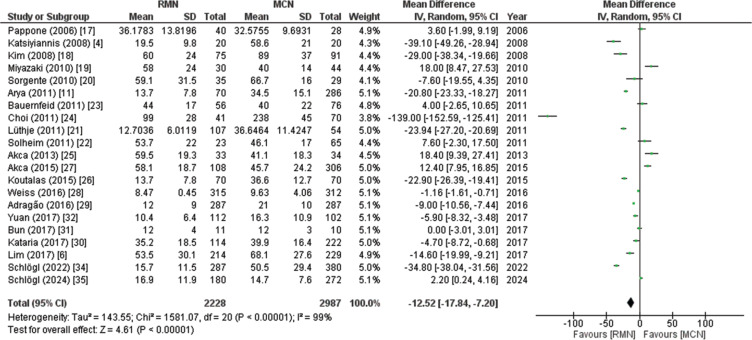
Forest plot of fluoroscopy time. This figure shows a pooled analysis of 22 studies, revealing that patients who underwent remote magnetic navigation had 12.52-min shorter fluoroscopy times than patients who underwent manual catheter navigation. *Abbreviations:* CI, confidence interval; IV, inverse variance; MCN, manual catheter navigation; MD, mean difference; RMN, remote magnetic navigation; SD, standard deviation.

Twenty-one studies including 5215 patients (RMN, 2228; MCN, 2987) reported the fluoroscopy time. The pooled analysis showed that the fluoroscopy time of the RMN group was significantly shorter as compared to that of the control group treated with the MCN approach. The use of RMN decreased the fluoroscopy time by a mean duration of 12.52 min (MD, −12.52; 95% CI, −17.84 to −7.20; *P* < .00001), as illustrated in **Figure 4**. Due to high heterogeneity (*I*^2^ = 99%), a leave-one-out sensitivity analysis was performed, and the result seemed to be non-significant. The subgroup analysis showed that the fluoroscopy time was significantly shorter (*P* = .02 and *P* = .001, respectively) among individuals with a history of DM (MD, −21.09; 95% CI, −29.87 to −12.32; *P* < .00001) and CAD (MD, −26.50; 95% CI, −37.75 to −15.26; *P* < .00001) in contrast to those without a history of DM (MD, −6.34; 95% CI, −14.93 to 2.24; *P* = .15) or CAD (MD, −5.59; 95% CI, −11.67 to 0.48; *P* = .07), indicating that these are potential causes of heterogeneity in the studies reporting this outcome. The GRADE strength of the evidence was very low, as displayed in **Supplementary Table S4**.

##### Total complication rate

**Figure 5:**
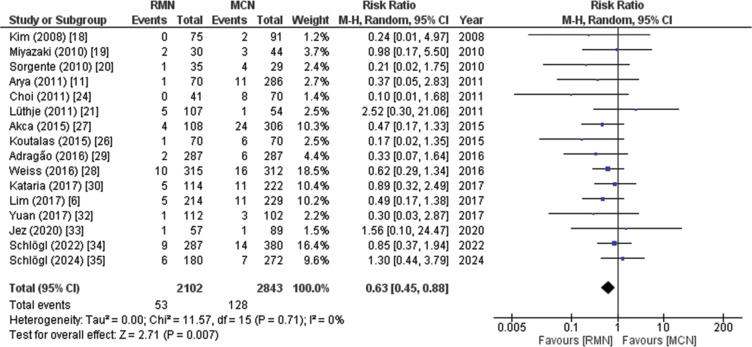
Forest plot of total complication rate. This figure presents a pooled analysis demonstrating a statistically significant decrease in complication rates for patients who underwent remote magnetic navigation compared to patients who underwent manual catheter navigation. *Abbreviations:* CI, confidence interval; M–H, Mantel–Haenszel; MCN, manual catheter navigation; RMN, remote magnetic navigation; RR, relative risk.

Sixteen studies including 4945 patients (RMN, 2102; MCN, 2843) reported the total complication rate. The pooled analysis showed a significantly lower total complication rate in patients who underwent the RMN procedure compared to those in the control group who underwent the MCN procedure (RR, 0.63; 95% CI, 0.45–0.88; *P* = .007), as illustrated in **Figure 5**. The absolute pericardial effusion rates were 0.6% in the RMN group and 2% in the MCN group, contributing significantly to the overall complication rate. Notably, only 4 out of the 22 included studies (18.18%) reported the use of contact force–sensing catheters in the MCN group, which may have influenced the observed complication rates.^31–33,35^ The heterogeneity in catheter technology across studies underscores the need for future comparisons using contemporary ablation tools. Furthermore, esophageal complications, such as esophageal injury or atrio-esophageal fistula, were not reported in the included studies. The GRADE strength of the evidence was low, as displayed in **Supplementary Table S4**.

#### Secondary outcomes

The secondary outcomes we evaluated in our study were acute success rate, pericardial effusion with or without tamponade, vascular access complications, AF recurrence rate, ablation time, and RF application duration.

##### Acute success rate

The pooled analysis of 10 studies including 2239 patients (RMN, 931; MCN, 1308) showed that there was no statistically significant difference in the acute success rate between the RMN group and the control group using the MCN method (RR, 0.93; 95% CI, 0.86–1.01; *P* = .07), as illustrated in **Table 3**. The GRADE strength of the evidence was low, as displayed in **Supplementary Table S4**.

##### Pericardial effusion with or without tamponade

Fifteen studies including 4517 patients (RMN, 1993; MCN, 2524) reported pericardial effusion with or without tamponade. The pooled analysis revealed that the incidence of pericardial effusion was significantly lower in the RMN group as opposed to the control group employing the MCN procedure (RR, 0.44; 95% CI, 0.23–0.82; *P* = .010), as illustrated in **Table 3**. The GRADE strength of the evidence was high, as displayed in **Supplementary Table S4**.

##### Vascular access complications

Twelve studies including 3827 patients (RMN, 1559; MCN, 2268) reported vascular access complications. The pooled analysis showed that there was no significant difference between the RMN group and the control group treated with the MCN method in terms of the incidence of vascular access complications (RR, 0.93; 95% CI, 0.46–1.89; *P* = .85), as illustrated in **Table 3**. The GRADE strength of the evidence was low, as displayed in **Supplementary Table S4**.

##### Atrial fibrillation recurrence rate

Fifteen studies including 3996 patients (RMN, 1748; MCN, 2248) reported the AF recurrence rate. The pooled analysis showed that there was no significant difference between the RMN group and the control group using the MCN approach in terms of the AF recurrence rate (RR, 0.97; 95% CI, 0.88–1.07; *P* = .59), as illustrated in **Table 3**. The GRADE strength of the evidence was low, as depicted in **Supplementary Table S4**.

##### Ablation time

Five studies including 677 patients (RMN, 339; MCN, 338) reported the ablation time. The pooled analysis revealed that the ablation time of the RMN group was significantly longer than that of the control group treated with MCN ablation. The use of RMN increased the ablation time by a mean duration of 25.80 min (MD, 25.80; 95% CI, 7.28–44.32; *P* = .006), as illustrated in **Table 3**. The GRADE strength of the evidence was very low, as displayed in **Supplementary Table S4**.

##### Radiofrequency application duration

Ten studies including 3195 patients (RMN, 1264; MCN, 1931) reported the RF application duration. The pooled analysis showed that the RF application duration of the RMN group was significantly prolonged in comparison to the control group managed with the MCN modality. The use of RMN increased the RF application duration by a mean of 14.67 min (MD, 14.67; 95% CI, 7.58–21.76; *P* < .0001), as illustrated in **Table 3**. The GRADE strength of the evidence was very low, as displayed in **Supplementary Table S4**.

##### Meta-regression and Egger’s regression analysis

Meta-regression analysis was performed to explore the source of high heterogeneity in the primary outcomes, including freedom from AF (*P* < .00001; *I*^2^ = 73%), procedure time (*P* < .00001; *I*^2^ = 96%), and fluoroscopy time (*P* < .00001; *I*^2^ = 99%), considering sample size and age, which demonstrated that these two factors are not the causes of high heterogeneity. Moreover, we conducted Egger’s test to detect publication bias in the primary outcomes, including freedom from AF, procedure time, fluoroscopy time, and total complication rate. The analysis showed non-significant intercepts for all primary outcomes (*P* = .76, *P* = .98, *P* = .74, and *P* = .15, respectively), suggesting no evidence of publication bias in these outcomes, as illustrated in **Supplementary Table S5**.

## Discussion

Catheter ablation for AF is a complex procedure with less-than-ideal long-term success rates, notable risks of major complications, and considerable radiation exposure. Recently, RMN systems have been developed, which provide increased precision and enhanced stability in catheter–tissue contact. As there is significant interest in the potential advantages of RMN systems, we performed a meta-analysis to compare the safety and efficacy of RMN and MCN in AF ablation.

This study found that RMN and MCN were similarly effective in achieving freedom from AF (RR, 0.94; *P* = .23). Meanwhile, RMN was linked to a lower overall complication rate (RR, 0.63; *P* = .007), a longer procedure time (by 48.58 min) (MD, 48.58; *P* < .00001), and a shorter fluoroscopy time (by 12.52 min) (MD, −12.52; *P* < .00001). However, upon subgroup analysis, it was found that the procedural time was significantly increased in patients with prior AF, demonstrating that AF is a source of heterogeneity in the included studies. Furthermore, the presence and absence of DM and CAD significantly contributed to the heterogeneity in the fluoroscopy time. The observed heterogeneity in fluoroscopy times among patients with DM and CAD may be attributed to the complexity of ablation procedures in these populations. Patients with DM and CAD often have more advanced atrial remodeling, larger left atrial sizes, and comorbid conditions that can prolong procedural times and increase the need for fluoroscopic guidance. Some research has highlighted several procedural advantages of RMN, suggesting that, as this technique gains popularity, its success rate could improve. The greater maneuverability and precise control of the catheter tip enable it to access difficult areas, potentially leading to more successful ablations in previously challenging cases.^11,26,36^ Furthermore, RMN provides better energy delivery from the catheter tip due to more stable and continuous contact at the ablation site.^37^ Conversely, MCN catheters face limitations due to their predefined curves, making it difficult to reach certain heart regions and maintain stability while delivering sufficient RF energy. Despite these procedural differences, our study indicated that AF recurrence rates were comparable between the two groups. This may be attributed to the similar methods used for monitoring AF recurrence and resolution in both groups. Additionally, undetected asymptomatic AF recurrences could be a factor, suggesting that future studies might benefit from longer follow-up periods to further evaluate efficacy.

Although RMN offers numerous advantages over MCN, our analysis indicates that RMN still requires longer overall procedure times. This is likely due to the limited experience and exposure physicians have with this technique. As practitioners become more proficient with RMN, it is expected that the procedure duration will decrease, making RMN more cost-effective when performed by skilled hands. Kim et al. demonstrated that an increase in the number of RMN procedures performed by a provider correlated with a reduction in operative time.^18^ Prolonged procedures not only increase costs in the health care sector but also impose physical strain on providers and staff due to the extended use of heavy lead protection. However, some institutions mitigate this by positioning the electrophysiologist outside the operative suite, thereby shielding them from the burden of heavy protective gear. The most significant finding of this study was the notably reduced fluoroscopy time in RMN procedures. This reduction enhances the safety of patients, operators, and support staff. The primary reason for the decreased use of fluoroscopy is likely the superior catheter stability and secure tissue contact that RMN offers compared to MCN techniques.^21^ Additionally, during RMN ablation, the electrophysiologist can be positioned outside the operative laboratory with protective barriers, thereby reducing fluoroscopy exposure while remotely controlling the catheter. RMN, though costlier due to advanced equipment, reduces fluoroscopy time, improves energy efficiency, and enhances operator safety by minimizing radiation exposure. As costs decrease with technological advancements, its cost-effectiveness is expected to improve, supporting its clinical adoption.^38^ However, it is important to recognize that advancements in MCN techniques, such as the zero-gravity shielding system and fluoroless workflows, along with the development of new-generation contact force–sensing catheters and steerable sheaths, may diminish the theoretical benefits of RMN. Further studies are necessary to compare these latest MCN catheters with RMN. The reduced fluoroscopy time associated with RMN is a significant advantage, particularly in centers where fluoroless workflows are not yet standard practice. However, it is important to note that fluoroless workflows for RMN are less commonly reported due to the reliance on fluoroscopy for initial catheter positioning and navigation in the magnetic field. While intracardiac echocardiography (ICE) can be used to reduce fluoroscopy, it is not routinely integrated into RMN workflows.^39,40^ Future advancements in RMN technology, such as improved integration with ICE or three-dimensional mapping systems, may facilitate fluoroless workflows and further enhance the safety profile of RMN.

Pulmonary vein isolation (PVI) ablation is associated with a range of complications. The most common issues associated with PVI include vascular access site complications and pericarditis, but more severe complications such as atrio-esophageal fistula, cardiac tamponade, and even death can occur.^41^ In our analysis, the MCN group exhibited a higher overall complication rate, including incidents of pericardial effusion with or without tamponade. This may be due to the greater force required for effective endocardial contact with MCN catheters, which are known to have inferior tissue contact.^19^ Conversely, the flexibility of RMN catheters results in less trauma, reducing the risk of myocardial inflammation and perforation.^19^ However, vascular access complications were similar between the two groups, as the techniques for obtaining vascular access do not differ between catheter types. The techniques for vascular access closure were not reported.

The learning curve for RMN is an important consideration for its clinical adoption. Studies suggest that operators typically require 12–50 cases to achieve proficiency with the RMN system, after which procedure times and fluoroscopy use decrease significantly.^17,18^ As operators gain experience, they become more efficient, which can improve outcomes and reduce costs. This learning curve highlights the importance of training and experience in maximizing the benefits of RMN. Future studies should evaluate the impact of operator experience on RMN outcomes to better understand its role in clinical practice.

While RMN offers several advantages, including reduced fluoroscopy time and improved catheter stability, it is important to acknowledge the evolving landscape of AF ablation. Recent advancements in MCN, such as the use of contact force-sensing catheters, steerable sheaths, and high-power/short-duration ablation strategies, have improved procedural outcomes and safety.^42,43^ Additionally, the emergence of pulsed field ablation (PFA) as a novel energy source has further revolutionized AF ablation, offering shorter procedure times and potentially lower complication rates.^44,45^ These developments highlight the need for ongoing comparisons between RMN and modern MCN techniques, as well as emerging technologies such as PFA, to determine their relative efficacy and safety in contemporary clinical practice

### Limitations

This study has several limitations. Most of the included studies were prospective, non-randomized controlled trials. The follow-up period varied significantly, ranging from immediately post-procedural to 44 months; shorter follow-up durations in some studies might have impacted the results. The heterogeneity in ablation strategies, patient characteristics (eg, paroxysmal versus persistent AF, left atrial size), and outcome measures across studies also complicates the interpretation of success rates; for instance, the inclusion of studies using outdated techniques or non-contact force catheters in the MCN group may have influenced the observed success rates, making them less reflective of modern ablation practices. Additionally, the variability in follow-up durations and monitoring methods (eg, intermittent ECG vs. continuous Holter monitoring) further limits the comparability of results. Furthermore, the methods for detecting and defining recurrence were not standardized and differed among the studies, with some considering any recurrence of arrhythmias (including AF, atrial flutter, or atrial tachycardia) and others only considering AF recurrence lasting >60 s. However, a subgroup analysis of studies with follow-up periods of ≥12 months, using Holter monitors to detect AF, showed no difference in AF recurrence rates, making this a less-significant limitation.^20–22,26,28,29,32^ The definitions of procedural complications were not detailed in all studies. Additionally, the influence of anti-arrhythmic drugs on the study results could not be assessed, as a subgroup analysis of patients with and without anti-arrhythmic drug use was not possible. Moreover, the heterogeneity in ablation strategies and catheter technologies across the included studies limits the generalizability of our findings. For instance, only a minority of studies in the MCN group used contact force–sensing catheters, and no study used high-power/short-duration ablation strategies, which are now standard in modern AF ablation.^42,43^ This may have influenced the observed complication rates and procedural outcomes, making our results less reflective of contemporary MCN practices. Furthermore, the rapid adoption of PFA as a novel energy source for AF ablation underscores the need for future comparisons between RMN, modern MCN, and PFA to determine their relative efficacy and safety in the current era.^44,45^

While this study provides a comprehensive comparison of RMN and MCN, it is important to acknowledge the rapid advancements in ablation technologies that have emerged in recent years. Techniques such as high-power/short-duration ablation, single-shot devices, and PFA are revolutionizing the field of AF ablation, offering shorter procedure times and potentially lower complication rates.^42–45^ Additionally, advancements in RMN technology, such as the “Ablation History” software and improved magnetic catheter designs, may further enhance the precision and safety of RMN procedures. Future studies should compare RMN with these emerging technologies to determine their relative efficacy and safety in contemporary clinical practice.

Another limitation of this study is that 3 out of the 13 studies^4,31,35^ reporting acute success rate as an outcome were excluded from the forest plot analysis. In these studies, all patients in both the RMN and MCN groups achieved acute success (100% success rate), resulting in “not estimable” effect sizes in Review Manager (RevMan). While this exclusion may introduce some bias, it was necessary to ensure the accuracy and interpretability of the pooled analysis, as studies with 100% success rates in both groups do not contribute to the calculation of RR.

## Conclusion

In conclusion, our systematic review and meta-analysis suggest that RMN ablation may offer reduced fluoroscopy time and lower complication rates compared to MCN ablation, despite longer procedure times and similar freedom from AF rates. Further research is warranted to confirm these findings and elucidate the mechanisms underlying the observed differences in treatment outcomes. In addition, future research should focus on comparing RMN with contemporary MCN techniques, including contact force–sensing catheters and high-power/short-duration ablation, as well as emerging technologies such as PFA. Additionally, cost-effectiveness analyses are needed to inform the adoption of RMN in routine clinical practice. Ultimately, optimizing the selection of ablation techniques based on patient characteristics and procedural factors may help improve the effectiveness of AF management strategies and enhance patient outcomes.
